# Lobar analysis of classical and deep learning radiomic features in chronic airflow limitation

**DOI:** 10.1186/s12880-026-03020-9

**Published:** 2026-10-31

**Authors:** Emelie Bäcklin, Birgitta Janerot-Sjoberg, Örjan Smedby, Magnus Sköld, Eva Breznik

**Affiliations:** 1 Department of Clinical Science, Intervention and Technology (CLINTEC), Karolinska Institutet, Stockholm, Sweden; 2 Department of Biomedical Engineering and Health Systems, KTH Royal Institute of Technology, Stockholm, Sweden; 3 Department of Clinical Physiology, Karolinska University Hospital, Stockholm, Sweden; 4 Department of Medicine, Karolinska Institutet, Solna, Stockholm, Sweden; 5 Department of Respiratory Medicine and Allergy, Karolinska University Hospital, Stockholm, Sweden

**Keywords:** Radiomics, CT, Deep learning, Chronic airflow limitation

## Abstract

**Background:**

Quantitative chest computed tomography (CT) enables detailed assessment of structural lung abnormalities in chronic airflow limitation (CAL). We evaluated whether lobe-based classical radiomics and frozen deep learning transfer features from inspiratory and expiratory CT improved discrimination of CAL compared with handcrafted quantitative measures.

**Methods:**

In this population-based study, 2,579 participants underwent inspiratory and expiratory CT and post-bronchodilator spirometry. CAL, defined as post-bronchodilator FEV₁/FVC < 0.70, was the primary modeling endpoint and was present in 430 participants; 2,149 participants without CAL served as controls. Lung lobes were segmented using nnU-Net. Classical radiomic features were extracted with PyRadiomics, and deep learning transfer features were obtained from frozen 3D U-Net and SegResNet encoders pretrained on the non-lung AbdomenAtlas dataset. A previously developed handcrafted feature set was included as a reference. Elastic-net regularized logistic regression with 10-fold cross-validation was used for classification, and performance was assessed by the area under the receiver operating characteristic curve (AUC).

**Results:**

Models based on expiratory features outperformed inspiratory-only models (AUC improvement 0.03–0.08). The best performance was achieved by classical radiomics combining inspiratory and expiratory features (AUC 0.87; positive predictive value 49%; negative predictive value 95%), exceeding the handcrafted model (AUC 0.81; PPV 34%; NPV 94%). Frozen transfer features from the non-lung-pretrained encoders showed slightly lower discrimination than classical radiomics, and combining feature types did not improve performance.

**Conclusion:**

Lobe-based classical radiomics from combined inspiratory and expiratory CT improved cross-validated discrimination of spirometry-defined CAL in this population-based cohort, with expiratory imaging contributing substantially to optimal performance.

**Supplementary Information:**

The online version contains supplementary material available at https://doi.org/10.1186/s12880-026-03020-9.

## Background

Quantitative analysis of computed tomography (CT) images has become increasingly important, with radiomics providing a high-throughput approach for characterizing structural and tissue properties beyond visual assessment. Introduced by Lambin et al. in 2012 [1], radiomics has been widely applied in oncology [2] and increasingly in infectious and inflammatory lung diseases, including COVID-19 [3, 4], as well as in the identification [5–7] and staging [8, 9] of chronic obstructive pulmonary disease (COPD).

Radiomic features can be extracted using classical methods or deep learning (DL). Classical radiomics relies on predefined mathematical descriptors of intensity, shape, and texture, whereas DL-derived features represent image characteristics learned during network training. Advances in DL have transformed medical imaging across CT, magnetic resonance imaging (MRI), and ultrasound, supporting detection, segmentation, and classification tasks [10]. DL features may capture complex, hierarchical patterns beyond predefined descriptors, although they are generally less interpretable than established quantitative CT measures such as lung volume and low-attenuation volume percentage. These representations may nevertheless provide complementary information. Through transfer learning, pretrained networks can either be fine-tuned for a target task or used as fixed feature extractors without updating their weights [11].

COPD is a leading cause of death worldwide, affecting more than 400 million people [12], and increasing attention is directed toward early detection and intervention [13]. It is a heterogeneous condition with substantial variability in risk factors, symptoms, structural abnormalities, and prognosis. Chronic airflow limitation (CAL), defined by persistent airflow obstruction after bronchodilation, is a central physiological characteristic of COPD and provides an objective measure of pulmonary function within this heterogeneous disease. In this study, CAL was used as the primary modeling endpoint, while COPD was considered a symptomatic subgroup of participants with CAL. Individuals with CAL but without respiratory symptoms may represent asymptomatic or subclinical airflow obstruction. Focusing on CAL therefore enables evaluation of CT-derived features against an objective physiological marker, including early or mild airflow obstruction that may not yet have been identified as COPD.

Quantitative CT provides a means of characterizing structural lung abnormalities associated with airflow limitation, including emphysema, airway remodeling, and air trapping [14]. Although the clinical relevance of expiratory CT has been recognized since 2018 [15], its systematic evaluation in large population-based cohorts remains limited. Advances in automated segmentation of the five lung lobes has enabled reproducible regional analysis in large datasets [16]. Lobar quantitative analysis has been applied to pulmonary function assessment [17], emphysema quantification [18], lung cancer risk assessment [19], and COPD staging [9]. Compared with whole-lung analysis, a lobe-based approach may better capture the regional heterogeneity of structural and functional lung abnormalities.

The present study aimed to evaluate whether lobe-based classical radiomic and DL-derived features from inspiratory and expiratory CT improve discrimination of CAL compared with previously developed handcrafted quantitative CT measures [20]. Classical radiomic features were extracted using PyRadiomics, while frozen DL transfer features were obtained from two pretrained convolutional neural network architectures, SegResNet and 3D U-Net. These architectures represent different design principles, with SegResNet employing residual connections to enhance feature propagation and 3D U-Net using a multiscale encoder–decoder structure with skip connections to preserve spatial information. The handcrafted reference model included total and lobar measures of lung volume and low-attenuation volume from inspiratory and expiratory CT. By comparing these feature representations individually and in combination, the study evaluated the potential contribution of lobe-based radiomics and frozen transfer features to CAL discrimination.

We hypothesized that combining inspiratory and expiratory CT with lobe-based classical radiomic and DL-derived features would improve discrimination of CAL compared with conventional handcrafted quantitative CT measures.

## Materials and methods

### Dataset

Participants were recruited from the Stockholm subset of the Swedish CArdioPulmonary bioImage Study (SCAPIS), a population-based, cross-sectional, multicentre cohort including men and women aged 50–64 years [21], conducted between 2013 and 2018. The SCAPIS study was conducted in accordance with the principles of the Declaration of Helsinki (1975), and all participants provided written informed consent. Data available from SCAPIS included inspiratory and expiratory chest CT scans, questionnaire information, and dynamic spirometry measurements.

Participants were classified according to post-bronchodilator dynamic spirometry using the ratio of forced expiratory volume in 1 s (FEV₁) to forced vital capacity (FVC). The primary modeling endpoint was chronic airflow limitation (CAL), defined as post-bronchodilator FEV₁/FVC < 0.70. Individuals were assigned to either a non-CAL group (FEV₁/FVC ≥ 0.70) or a CAL group (FEV₁/FVC < 0.70). Among participants with CAL, those reporting respiratory symptoms, including dyspnea, cough, phlegm production, or wheezing, were classified as a symptomatic COPD subgroup. COPD status, including Global Initiative for Chronic Obstructive Lung Disease (GOLD) stage, was used to describe the clinical characteristics of the CAL group but was not used as a separate modeling endpoint. GOLD stage was assigned according to the severity of airflow limitation based on post-bronchodilator FEV₁ expressed as a percentage of the predicted value. CAL participants without reported respiratory symptoms were retained in the CAL group and interpreted as having asymptomatic or subclinical airflow obstruction [22]. Information on smoking status and chronic bronchitis, defined as cough and phlegm lasting at least three months in two consecutive years, was obtained from self-reported questionnaires. Emphysema was determined by visual assessment performed by a radiologist.

Out of the 5,038 individuals in the SCAPIS Stockholm cohort, a total of 3,385 participants satisfied the initial inclusion criteria, defined as the availability of adequate inspiratory and expiratory CT scans, dynamic spirometry, and questionnaire data. Sixty cases were reserved for training of the lobe segmentation algorithm, and forty participants were excluded due to image-processing errors. All 430 participants with CAL were included. Participants without CAL were then selected by sex-stratified random sampling at an approximate 5:1 non-CAL ratio (*n* = 2,149), resulting in a total analytical cohort of 2,579 participants (1,328 men). Of these, 296 had been included in a previous publication [23]. Details on inclusion are provided in Additional file 1.

Characteristics of the participants are presented in Table 1. Among the 430 participants classified as having chronic airflow limitation (CAL), 179 met the criteria for COPD, of whom 148 (83%) had a FEV₁ ≥ 80% predicted, corresponding to mild airflow limitation.

**Table 1:** Participant characteristics, *n* = 2,579

Characteristics	non-CAL			CAL	
n	2149		(83%)	430	(17%)
Male	1074		(50%)	254	(59%)
Age, years	57		(53–61)	59	(55–63)
FEV_1_, L	3.3		(2.8–4.0)	3.0	(2.4–3.6)
FVC, L	4.3		(3.6–5.1)	4.7	(3.7–5.6)
FEV_1_/FVC	0.78		(0.75–0.81)	0.66	(0.63–0.68)
COPD	n/a		n/a	179	(42%)
GOLD stage:					
1	n/a		n/a	148	(83%)
2	n/a		n/a	25	(14%)
3	n/a		n/a	5	(2.8%)
4	n/a		n/a	1	(0.56%)
Current smokers, n	226		(11%)	101	(24%)
Pack years	11		(10–21)	30	(19–40)
Previous smokers, n	880		(41%)	188	(44%)
Pack years	10		(3.8–20)	12	(4.0–23)
Never-smokers, n	1043		(48%)	141	(33%)
Chronic bronchitis	89		(4.1%)	38	(8.8%)
Emphysema, n	117		(5.4%)	73	(17%)

Note: Values are presented as median (interquartile range) or frequency (percentage). GOLD-stage percentages were calculated among participants in the symptomatic COPD subgroup (*n* = 179). Pack-years are reported separately for current and former smokers

### Computed tomography of the lung (Chest CT)

Chest CT images were acquired during breath-hold at maximal inspiration and expiration using a dual-source CT scanner (Somatom Definition Flash; Siemens Healthineers). Scans were performed from the lung apex to the base in the supine position, without the use of contrast media or bronchodilators. Participants received coaching and practiced breath-holding at full inspiration and expiration. Acquisition parameters included a detector configuration of 128 × 0.6 mm, tube voltage of 120 kVp, CARE Dose4D quality reference mAs of 25, rotation time of 0.5 s, pitch of 0.9, and a matrix size of 512 × 512. Reconstruction parameters were a slice thickness of 0.75 mm, increment of 0.6 mm, and I50f medium-sharp lung reconstruction kernel. The field of view was adjusted to ensure complete lung coverage. The median effective radiation dose was 1.7 mSv for inspiratory scans and 1.5 mSv for expiratory scans.

### Pulmonary function tests (PFTs)

Dynamic spirometry (Jaeger Spirometer™, Vyaire Medical, USA) was conducted according to clinical routine 15 min after inhalation of 400 µg salbutamol, with participants seated and wearing a nose clip. Forced expiratory volume in 1 s (FEV₁) and forced vital capacity (FVC) were measured in accordance with ATS/ERS standards [24].

### Lung lobe segmentation

Lung lobe segmentation was performed using version 2 of the DL framework nnU-Net, which was selected for its automated configuration and robust performance in medical image segmentation [25]. Images from 60 participants were manually annotated by an experienced radiologist using MiaLab (https://mialab.org), an in-house medical image analysis software. Model training was conducted using standard nnU-Net settings, including the Adam optimizer with a learning rate of 0.01, Dice–cross-entropy loss, 1,000 training epochs, and a batch size of 2. Of the 60 image pairs, 48 were used for training, 11 for evaluation, and one was excluded due to processing errors. The mean Dice similarity coefficient for the evaluation set was 96% (LU 97%, LL 97%, RU 97%, RM 94%, RL 97%).

### Feature extraction

#### Classical radiomics features

PyRadiomics (version 1.0.5) [26] was used to extract standardized first-order, shape, and texture features from each lung lobe in inspiratory and expiratory CT images. Feature extraction used a fixed bin width of 25, resampled voxel spacing of 1 × 1 × 1 mm, and sitkBSpline interpolation. Texture features included gray-level co-occurrence, run-length, size-zone, dependence, and neighboring gray-tone difference matrices. Further details are provided in the PyRadiomics documentation [27].

In addition to the original images, Laplacian of Gaussian (LoG) filtering was applied at σ = 1.0, 2.0, 3.0, 4.0, and 5.0 mm, and wavelet filtering (coif1, Coiflet family) generated eight frequency sub-bands (LLL, LLH, LHL, LHH, HLL, HLH, HHL, and HHH). In total, 1,316 radiomic features were extracted per lung lobe, yielding 13,160 features across all five lobes and both imaging phases.

#### Frozen transfer deep learning features

Deep learning transfer features were extracted using pretrained SegResNet and 3D U-Net encoders obtained from the publicly available SuPreM framework (https://github.com/MrGiovanni/SuPreM), trained on the AbdomenAtlas 1.1 dataset [28]. The pretrained weights were kept frozen without additional training or fine-tuning, and the networks were used solely as feature extractors. As pretraining was performed on abdominal rather than lung CT, the resulting representations were considered non-lung-pretrained transfer features.

CT images and lung masks were resampled to 1.5 × 1.5 × 1.5 mm voxel spacing. Intensities were clipped to − 1000 to 300 HU and linearly scaled to [0, 1]. Each lung lobe was cropped using a three-dimensional bounding box derived from its binary mask, with voxels outside the lobe set to zero.

Features were extracted from the frozen encoders in evaluation mode using global average pooling of encoder feature maps. For 3D U-Net, pooled activations from four successive downsampling levels were concatenated, yielding 960 features per lobe. For SegResNet, pooled activations from successive downsampling stages, the encoder bottleneck, and selected internal encoder blocks captured using forward hooks were concatenated, yielding 608 features per lobe.

#### Handcrafted features

In a previous study, a total of 48 total and lobar handcrafted features were extracted [20]. To assess lung volumes and identify CAL, these features included inspiratory and expiratory lung volumes, lung volume change (inspiratory volume minus expiratory volume), and the lung volume ratio (inspiratory volume divided by lung volume change). The proportion of low-attenuation volume was also assessed as a marker of structural lung changes and was quantified as the percentage of lung voxels below − 950 HU—an established threshold associated with pulmonary emphysema [29], and below − 856 HU which has been linked to the presence of air trapping [30]. All features were extracted from both the total lung and individual lung lobes, using inspiratory and expiratory CT images.

### Exploratory feature visualization

Feature-space structure was explored using dimensionality reduction for visualization purposes only. Uniform Manifold Approximation and Projection (UMAP), a non-linear dimensionality reduction method, was used to project high-dimensional feature data into a three-dimensional space for visual inspection.

Prior to visualization, features were robustly scaled and near-zero variance features were removed. For the classical radiomics and deep learning-derived feature sets, principal component analysis (PCA) was applied before UMAP to reduce dimensionality. Sixty principal components were retained for the classical radiomics features, explaining nearly all variance. For the U-Net and SegResNet feature sets, the retained components explained 77% and 74% of the variance, respectively. The handcrafted feature set was visualized without prior PCA because of its lower dimensionality.

All retained principal components were z-score standardized before UMAP embedding. UMAP was performed in Python version 3.13.7 using the umap-learn package version 0.5.9. Features were embedded into three dimensions using correlation distance, 50 nearest neighbors, a minimum distance of 0.7, and a fixed random state of 42. The UMAP analysis was not used for model training, feature selection, or performance evaluation.

### Regularized logistic regression

Binary classification of CAL was performed using regularized logistic regression with elastic-net regularization in GLMNET (α = 0.5). Elastic-net regularization was chosen to allow feature selection and coefficient shrinkage while improving stability in the presence of correlated, high-dimensional radiomic features. Logistic regression was used to obtain predicted probabilities in addition to binary classifications.

Feature inclusion was restricted to predefined feature classes. No supervised preselection, correlation filtering, near-zero variance filtering, PCA-based dimensionality reduction, or other outcome-informed feature selection was performed before cross-validation. Continuous features were standardized within the GLMNET framework. During 10-fold cross-validation, feature standardization and model fitting were performed using the training portion of each fold, and predicted probabilities were generated for the corresponding held-out fold. The regularization parameter λ was selected as the value that minimized the overall cross-validated error (λ_min). Model performance was evaluated using the resulting out-of-fold predicted probabilities. Thus, discrimination, precision–recall performance, and calibration were estimated from cross-validated predictions to reduce the risk of information leakage. PCA and UMAP were used only for visualization of feature-space structure and were not used for model training or performance estimation.

Model discrimination was assessed using receiver operating characteristic (ROC) analysis, and the area under the ROC curve (AUC) with 95% confidence intervals (CIs) calculated using DeLong’s nonparametric method was reported. Differences in AUC between models were assessed using DeLong’s test for correlated ROC curves, because model predictions were obtained for the same participants. Because CAL represented approximately 17% of the analytical cohort, precision–recall curves and the area under the precision–recall curve (AUC-PR) were also assessed. Calibration was evaluated using calibration plots and the Brier score.

For threshold-dependent metrics, a probability threshold was selected by maximizing the Youden index, defined as sensitivity + specificity − 1, using the pooled cross-validated out-of-fold predicted probabilities. Sensitivity, specificity, positive predictive value (PPV), and negative predictive value (NPV) were then calculated using this threshold. Because the threshold was selected and evaluated using the same set of out-of-fold predictions, these threshold-dependent metrics should be interpreted as internally optimized estimates and may retain some optimism. The Youden threshold was used for comparative model evaluation and was not intended to represent an independently validated or clinically optimized decision threshold. PPV and NPV were interpreted in relation to the CAL prevalence in the analytical cohort and may not directly generalize to populations with a different CAL prevalence.

Models were trained and evaluated separately using inspiratory features only, expiratory features only, and combined inspiratory and expiratory features. Classical radiomics, deep learning transfer features from SegResNet and 3D U-Net, and handcrafted features were analyzed individually and in combination.

### Statistical analysis

**Fig. 1:**
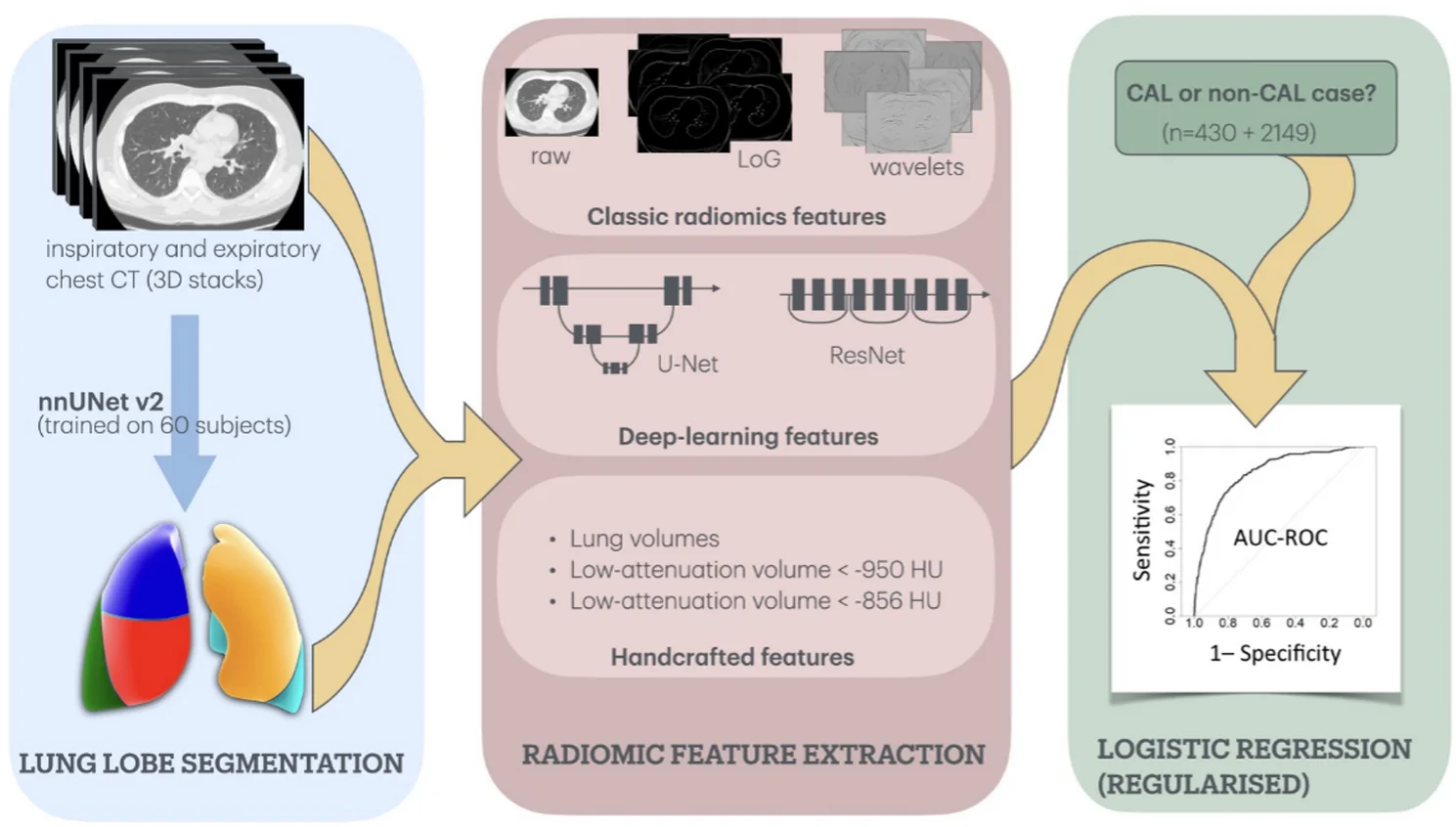
Workflow for lobe-based extraction of radiomic and handcrafted features to classify chronic airflow limitation (CAL)

Participant characteristics were summarized descriptively. The distributions of continuous variables were assessed visually using Q–Q plots; no formal hypothesis tests of normality were performed. Because the continuous variables showed departures from normality, they are presented as median (interquartile range), whereas categorical variables are presented as frequency (percentage). Statistical analyses were performed using R version 4.4.1 (R Core Team, Vienna, Austria). The overall study workflow is shown in Fig. 1.

## Results

### Regression models

**Fig. 2:**
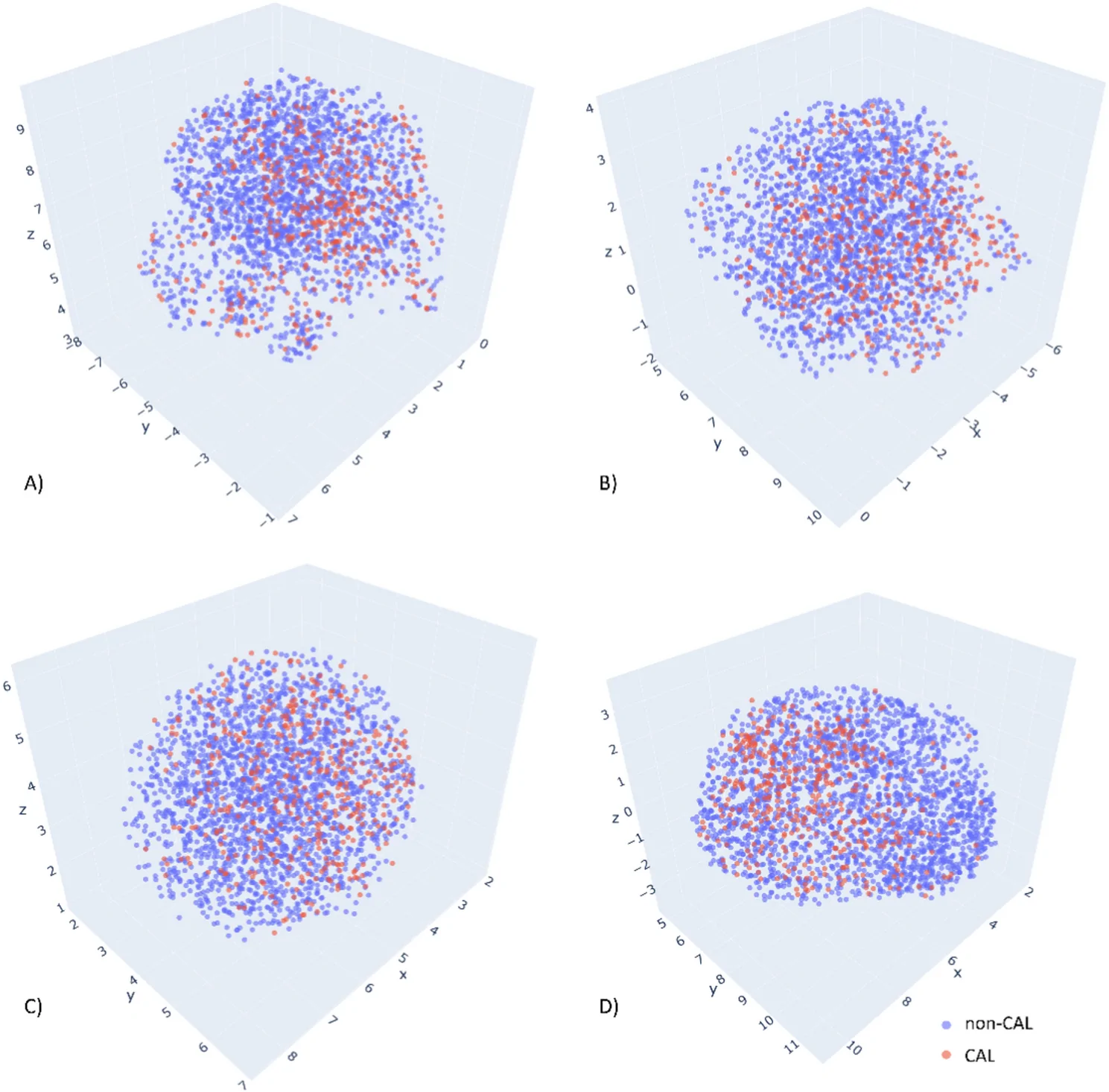
Exploratory Uniform Manifold Approximation and Projection (UMAP) visualization of feature representations. Panels show three-dimensional embeddings for (**A**) classical radiomics, (**B**) SegResNet, (**C**) 3D U-Net, and (**D**) handcrafted inspiratory–expiratory features. Blue indicates participants without chronic airflow limitation (non-CAL), and red indicates participants with chronic airflow limitation (CAL). Interactive three-dimensional visualizations are available at: https://github.com/emebac/Backlin_Radiomics_CAL_2026

UMAP visualization was used as an exploratory assessment of feature-space structure. Across all feature representations, participants with and without chronic airflow limitation showed largely overlapping distributions, with no clear or consistent separation between groups (Fig. 2). Interactive three-dimensional visualizations are available at the following repository: https://github.com/emebac/Backlin_Radiomics_CAL_2026.

**Fig. 3:**
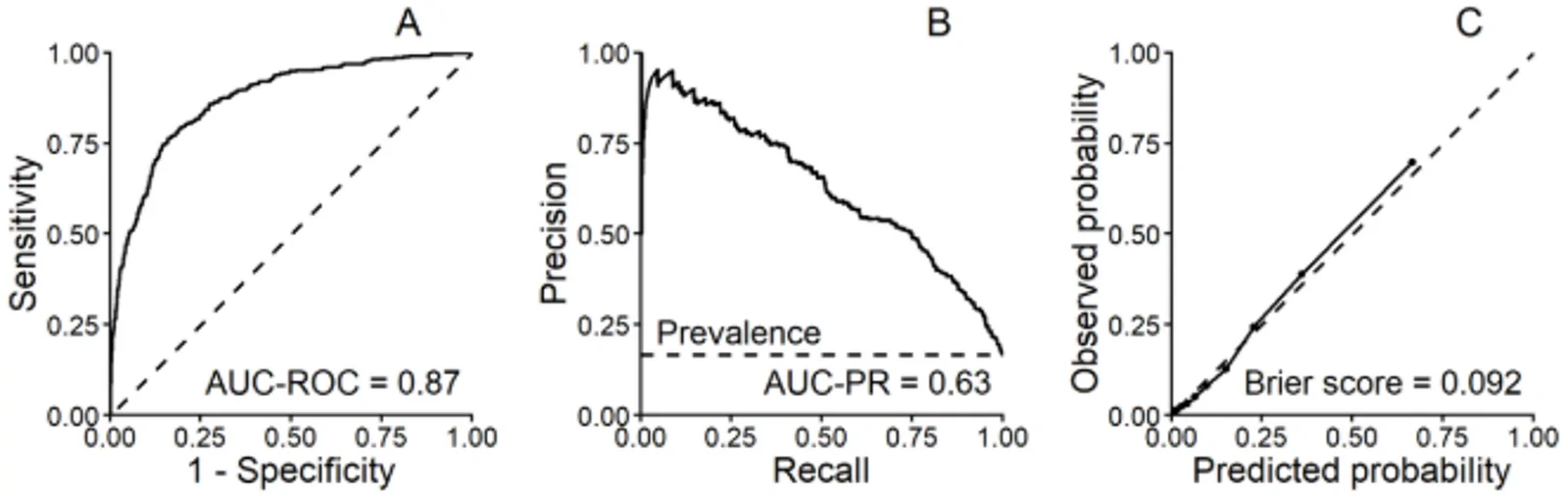
Performance of the combined inspiratory–expiratory classical radiomics model for chronic airflow limitation classification. (**A**) Receiver operating characteristic curve. (**B**) Precision–recall curve. (**C**) Calibration curve. Dashed lines indicate chance-level discrimination in A, CAL prevalence in B, and perfect calibration in C. The Brier score reflects the accuracy of predicted probabilities, with lower values indicating better performance. AUC-ROC, area under the receiver operating characteristic curve; AUC-PR, area under the precision–recall curve

Classical radiomics, SegResNet, and 3D U-Net models based on expiratory features consistently outperformed those based on inspiratory features, with absolute improvements in AUC ranging from 0.03 to 0.08 (Table 2). The best-performing model overall was the classical radiomics model combining inspiratory and expiratory features, which achieved significantly higher AUC than the frozen DL transfer features models according to DeLong’s test (*p* < 0.05). Full details of the pairwise AUC comparisons are provided in Additional file 2. Additional evaluation of the best-performing model showed an AUC-ROC of 0.87, an AUC-PR of 0.63, and a Brier score of 0.092 (Fig. 3). Corresponding ROC, precision–recall, and calibration plots for all presented models are provided in Additional file 3. For the best-performing model, the precision–recall curve remained above the prevalence line across most recall values, indicating improved precision relative to the CAL prevalence in the analytical cohort. The calibration plot showed approximate agreement between predicted and observed risks within the analytical cohort, with a Brier score of 0.092. External validation and potential recalibration are required. Compared with the expiratory-only model, the combined model improved the positive predictive value (PPV) by 8% points, rising from 41% to 49%, while the remaining performance metrics were largely unchanged. A similar pattern was observed for SegResNet, where combining inspiratory and expiratory features increased PPV from 35% to 41%. For the 3D U-Net model, performance was similar between the expiratory-only and combined feature models. The model based on handcrafted features achieved PPV values comparable to those of the other models but did not surpass them in terms of either AUC or PPV.

**Table 2:** Model metrics from regularized logistic regression using inspiratory (IN), expiratory (EX), and combined (IN + EX) features

Model		Sensitivity	Specificity	PPV	NPV	AUC (CI)
Classical	IN	74%	78%	40%	94%	0.83 (0.81–0.85)
	EX	84%	75%	41%	96%	0.86 (0.84–0.88)
	IN + EX	76%	84%	49%	95%	0.87 (0.85–0.89)
SegResNet	IN	71%	67%	30%	92%	0.75 (0.72–0.78)
	EX	82%	70%	35%	95%	0.83 (0.81–0.85)
	IN + EX	74%	78%	41%	94%	0.83 (0.81–0.86)
3D U-Net	IN	70%	71%	33%	92%	0.77 (0.75–0.80)
	EX	77%	74%	37%	94%	0.83 (0.81–0.85)
	IN + EX	82%	71%	36%	95%	0.84 (0.82–0.86)
Handcrafted, IN + EX		79%	69%	34%	94%	0.81 (0.78–0.83)

Analysis of the top 20 features ranked by the absolute magnitude of their coefficients in the final elastic-net model combining inspiratory and expiratory inputs revealed a strong predominance of expiratory features in the classical radiomics approach, with 16 expiratory features and 4 inspiratory features. In contrast, the frozen DL transfer feature models demonstrated a more balanced distribution: SegResNet selected 12 expiratory and 8 inspiratory features, while 3D U-Net showed the opposite pattern, with 12 inspiratory and 8 expiratory features. For classical radiomics, features extracted from the left and right lower lobes dominated (8 and 7 features respectively), while the distribution was more balanced for SegResNet and 3D U-Net. It is notable that the right middle lobe was only represented by one feature in each model.

In the classical radiomics model, all of the top 20 selected features originated from texture-based feature classes. The highest-ranked predictor was extracted from Laplacian of Gaussian (LoG)-filtered images (σ = 2.0 mm), and the second most important predictor was derived from wavelet-filtered images (LLL sub-band). Overall, 13 of the selected features were obtained from LoG-filtered images, six from wavelet-filtered images, and one from the original (unfiltered) images.

In the SegResNet model, the top 20 selected features were evenly distributed between representations extracted from earlier encoder stages and those derived from deeper encoder stages, reflecting contributions from both lower- and higher-level feature representations. Of the top 20 predictors identified by the 3D U-Net model, 12 were derived from the deepest encoder feature map. These findings describe the selected frozen transfer features and should not be interpreted as a general comparison between classical radiomics and lung-optimized deep learning models. Details on feature selection are provided in Additional file 4.

### Combined models

For combined features from classical radiomics, SegResNet, 3D U-Net and handcrafted (inspiratory and expiratory), a high NPV remained across all combinations. However, no improvement in AUC was observed compared with the best-performing individual model, and PPV improved only marginally by 1% point when combining 3D U-Net and handcrafted features; on the contrary, PPV decreased for combinations of classical radiomics with SegResNet and with 3D U-Net (Table 3). When combining classical radiomics and handcrafted features, all performance measures were preserved relative to the classical model based on inspiratory and expiratory features. Combining features from SegResNet and 3D U-Net resulted in only minor differences in performance.

**Table 3:** Metrics from models trained on combined features from inspiratory and expiratory images

Model	Sensitivity	Specificity	PPV	NPV	AUC (CI)
Classical + SegResNet	84%	75%	40%	96%	0.87 (0.85–0.89)
Classical + 3D U-Net	79%	81%	46%	95%	0.87 (0.86–0.89)
Classical + Handcrafted	77%	84%	49%	95%	0.87 (0.85–0.89)
SegResNet + Handcrafted	76%	78%	41%	94%	0.84 (0.82–0.86)
3D U-Net + Handcrafted	80%	73%	37%	95%	0.84 (0.82–0.86)

## Discussion

### Principal findings

In this population-based study, lobe-based classical radiomics combining inspiratory and expiratory CT achieved the highest discrimination of chronic airflow limitation (CAL), outperforming handcrafted features and frozen deep learning transfer features. Expiratory CT consistently provided greater discriminatory information than inspiratory CT alone, while combining different feature representations did not further improve performance. These findings highlight the potential value of expiratory imaging and lobe-based radiomics for identifying CAL.

### Relation to previous research

The greater discriminatory performance of expiratory CT is consistent with Feng et al. [6], who reported improved COPD identification and staging when expiratory imaging was added to inspiratory CT. However, unlike Feng et al. [6], who reported superior performance of deep learning, classical radiomics outperformed frozen deep learning transfer features in our study. This discrepancy may reflect differences in cohort composition, disease severity, and model optimization, particularly the use of non-lung-pretrained encoders without fine-tuning in our study.

Our best-performing model also achieved a higher AUC than reported by Guan et al. [7], whereas differences in PPV and NPV compared with Puchakayala et al. [5] may partly reflect variations in disease prevalence and cohort composition. Notably, most participants with symptomatic COPD in our cohort had GOLD stage 1 disease, highlighting the predominantly mild disease severity in this population-based sample.

### Interpretation of radiomic features

In the classical radiomics model, the highest-ranked predictors were exclusively texture features, predominantly derived from Laplacian of Gaussian (LoG)- and wavelet-filtered images, suggesting that spatial variations in lung attenuation may provide discriminatory information for CAL. These features may capture regional heterogeneity associated with small-airway dysfunction, air trapping, and early emphysematous changes, particularly on expiratory CT. The predominance of lower-lobe features further suggests that regional differences contribute to discrimination. However, these interpretations remain exploratory, and the selected features should be considered predictive model components rather than validated biological markers.

### Deep learning transfer features and feature fusion

Frozen deep learning transfer features showed lower discrimination than classical radiomics and did not improve performance when combined with it, possibly reflecting overlapping imaging information. However, the SegResNet and 3D U-Net encoders were pretrained on abdominal CT and used without lung-specific fine-tuning. Therefore, these findings reflect the performance of frozen, non-lung-pretrained representations rather than the broader potential of deep learning for CAL classification. Lung-specific pretraining, task-specific fine-tuning, and alternative feature-fusion strategies may yield different results.

### Clinical and technical implications

The improved discrimination achieved with expiratory CT suggests its potential value for identifying CAL. However, clinical implementation requires consideration of performance, computational efficiency, interpretability, radiation exposure, and technical reproducibility. Classical radiomics achieved the highest discrimination but required more extensive feature extraction and may be sensitive to variations in image acquisition and reconstruction. Frozen deep learning transfer features were computationally more efficient to extract but showed lower discrimination and interpretability.

A potential clinical workflow could combine automated lobe segmentation with a reduced set of validated, robust imaging features to identify individuals who may benefit from further clinical or spirometric assessment. However, external validation is required to establish its clinical utility. Paired inspiratory and expiratory CT also introduces additional radiation exposure, which could potentially be reduced using low-dose expiratory protocols, provided their diagnostic and radiomic performance is validated.

Technical factors affecting model generalizability include scanner type, radiation dose, image noise, reconstruction kernel and algorithm, breath-hold performance, and lobe segmentation variability. The standardized low-dose CT protocol and consistent reconstruction kernel used in this study reduced technical variability but may limit transferability to other imaging settings. Feature harmonization methods such as ComBat were not applied because no distinct scanner or reconstruction-protocol batches were present, but may be necessary in future multicenter or multi-protocol studies. Emerging technologies such as photon-counting CT may also influence feature stability and model transferability.

### Limitations

This study has several limitations. First, external validation was not available. The reported performance should therefore be interpreted as internally validated discrimination within the present population-based cohort rather than evidence of generalizable clinical performance. Validation in independent cohorts with different disease prevalences, disease severities, CT acquisition protocols, and population characteristics is required.

Second, a formal radiomic feature robustness analysis was not performed. Although elastic-net regularization reduces dimensionality and may limit overfitting, it does not establish the stability of individual selected features to variations in segmentation, preprocessing, resampling, discretization, or filtering. This consideration is particularly relevant for LoG- and wavelet-derived texture features. Accordingly, the selected features should be regarded as predictive model components rather than validated, biologically stable imaging biomarkers.

Third, the probability threshold used to calculate sensitivity, specificity, PPV, and NPV was selected from the pooled cross-validated out-of-fold predictions using the Youden index and evaluated using the same predictions. These threshold-dependent estimates may therefore retain some optimism and should be independently validated. Furthermore, PPV and NPV depend on disease prevalence and may differ substantially in populations with a different prevalence of CAL.

Fourth, CAL was defined primarily using the fixed post-bronchodilator FEV₁/FVC threshold of < 0.70. Although this definition is widely used and clinically straightforward, it does not account for age-related changes in lung function and may therefore result in some misclassification. An approach based on the lower limit of normal, incorporating age-, sex-, height-, and population-specific reference values, should be considered in future validation studies.

Finally, the deep learning encoders used in this study were pretrained on abdominal CT rather than chest CT and were applied without task-specific fine-tuning. The results therefore cannot be generalized to lung-specific or fully trained deep learning approaches.

### Future research

Future studies should externally validate these findings and directly compare lobe-based and whole-lung radiomics within the same population. Comparisons of imaging-only, clinical-only, and combined clinical-imaging models are also needed to determine the incremental value of radiomic features beyond established clinical and spirometric variables. Further work should assess radiomic feature robustness across segmentation methods, scanners, reconstruction protocols, and preprocessing settings. Lung-specific pretraining, self-supervised learning using chest CT, and supervised fine-tuning should also be investigated to determine whether task-optimized deep learning representations provide additional value beyond classical radiomics.

## Conclusion

In this population-based cohort, lobe-based classical radiomics combining inspiratory and expiratory CT achieved the highest internally validated discrimination of chronic airflow limitation, outperforming handcrafted features and frozen deep learning transfer features from non-lung-pretrained encoders. Expiratory CT consistently provided greater discriminatory information than inspiratory CT alone, highlighting its potential value for imaging-based characterization of airflow limitation. Combining frozen deep learning transfer features with classical radiomics did not improve performance, suggesting limited additional information from these representations in the present setting. External validation and assessment of radiomic feature robustness are required to establish the generalizability and potential clinical utility of these findings.

## Supplementary Information

Below is the link to the electronic supplementary material.

Supplementary Material 1: Additional file 1 (PDF). Participant inclusion flowchart. Abbreviations: CT, computed tomography; CAL, chronic airflow limitation; COPD, chronic obstructive pulmonary disease.

Supplementary Material 2: Additional file 2 (PDF). DeLong’s test for pairwise comparison of AUCs from the regression models. Presentation of p values for all pairwise AUC comparisons.

Supplementary Material 3: Additional file 3 (PDF). Model performance for all presented models, including receiver operating characteristic (ROC) curves, precision–recall curves, and calibration plots.

Supplementary Material 4: Additional file 4 (PDF). Selected features from the regularized logistic regression models. Tables and figures describing the features selected in the presented regression models.

## Data Availability

The data that support the findings of this study are available from the Swedish CardioPulmonaryBioImage Study (SCAPIS) but restrictions apply to the availability of these data, which were used under license for the current study, and so are not publicly available.

## Abbreviations

AUC: Area under the curve
CAL: Chronic airflow limitation
COPD: Chronic obstructive pulmonary disease
CT: Computed tomography
DL: Deep Learning
FEV₁: Forced Expiratory Volume in 1 s
FVC: Forced Vital Capacity
GOLD: Global Initiative for Chronic Obstructive Lung Disease
HU: Hounsfield units
LoG: Laplacian of Gaussian
NPV: Negative predictive value
PCA: Principal component analysis
PPV: Positive predictive value
ROC: Receiver operating characteristic
SCAPIS: Swedish CardioPulmonary bioImage Study
UMAP: Uniform Manifold Approximation and Projection
