# Supplementary material for: Lobar analysis of classical and deep learning radiomic features in chronic airflow limitation
Source: BMC Med Imaging. 2026 Oct 31;26:565. doi: 10.1186/s12880-026-03020-9 (PMC13723113; doi:10.1186/s12880-026-03020-9)
Supplement: Supplementary file 1 [file 12880_2026_3020_MOESM1_ESM.pdf]

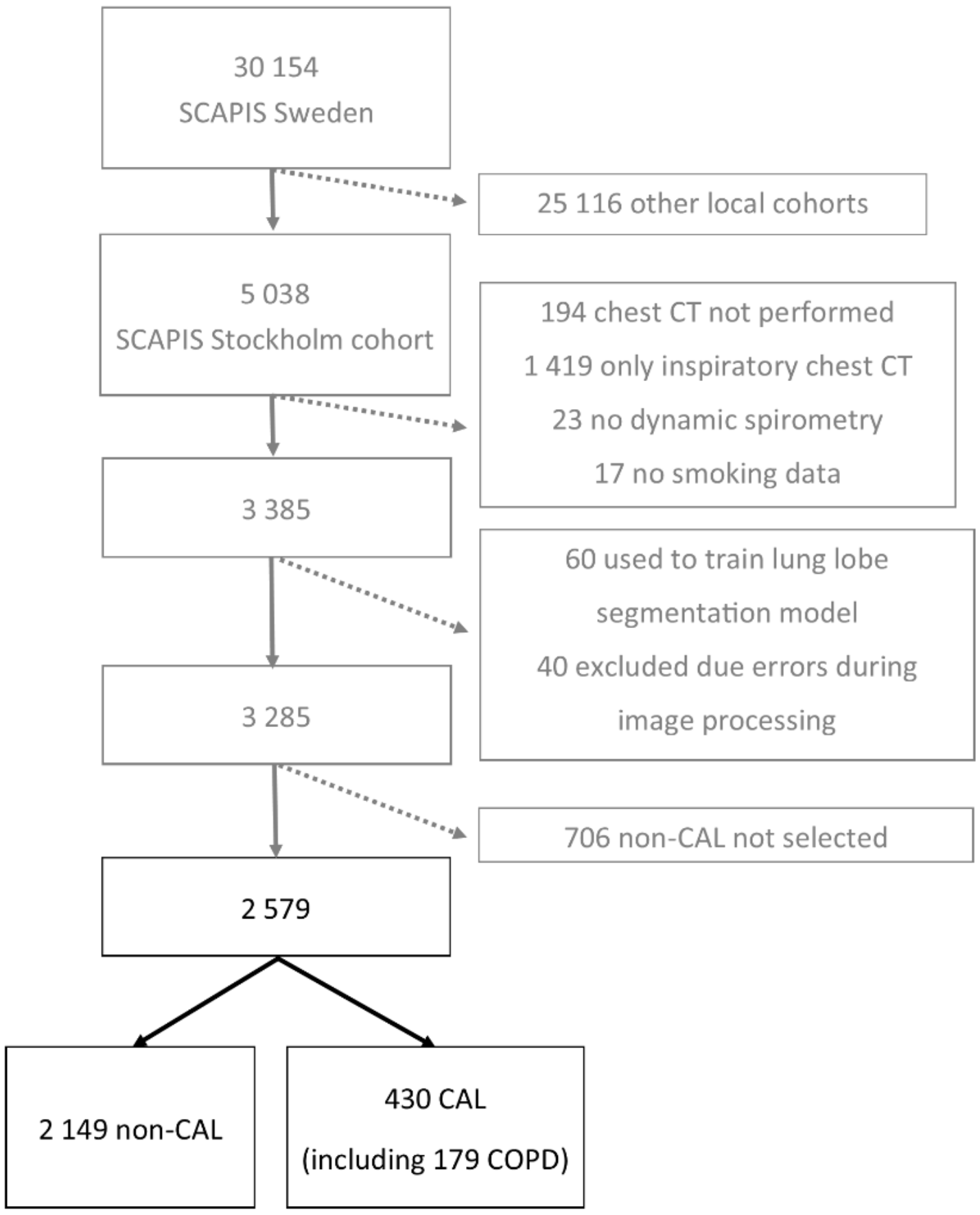

Figure 1. Participant inclusion. Abbreviations: CT, computed tomography; CAL, chronic airflow limitation; COPD, chronic obstructive pulmonary disease.
