# Supplementary material for: Lobar analysis of classical and deep learning radiomic features in chronic airflow limitation
Source: BMC Med Imaging. 2026 Oct 31;26:565. doi: 10.1186/s12880-026-03020-9 (PMC13723113; doi:10.1186/s12880-026-03020-9)
Supplement: Supplementary file 2 [file 12880_2026_3020_MOESM2_ESM.pdf]

Table 1. DeLongs test for comparison of AUC from regression models.

	Models	*p*
Classic	IN vs. EX	0.02
	IN vs. INEX	<0.001
	IN+EX vs. EX	0.50
SegResNet	IN vs. EX	<0.001
	IN vs. INEX	<0.001
	IN+EX vs. EX	0.50
3D U-Net	IN vs. EX	<0.001
	IN vs. INEX	<0.001
	IN+EX vs. EX	0.08
Intra-class	Classic IN+EX vs. 3D U-Net IN+EX	0.02
	Classic IN+EX vs. SegResNet IN+EX	0.01
	Classic IN+EX vs. Handcrafted	<0.001
	3D U-Net IN+EX vs. SegResNet IN+EX	0.50
	3D U-Net IN+EX vs. Handcrafted	<0.001
	SegResNet IN+EX vs. Handcrafted	0.002
