# Supplementary material for: Lobar analysis of classical and deep learning radiomic features in chronic airflow limitation
Source: BMC Med Imaging. 2026 Oct 31;26:565. doi: 10.1186/s12880-026-03020-9 (PMC13723113; doi:10.1186/s12880-026-03020-9)
Supplement: Supplementary file 3 [file 12880_2026_3020_MOESM3_ESM.pdf]

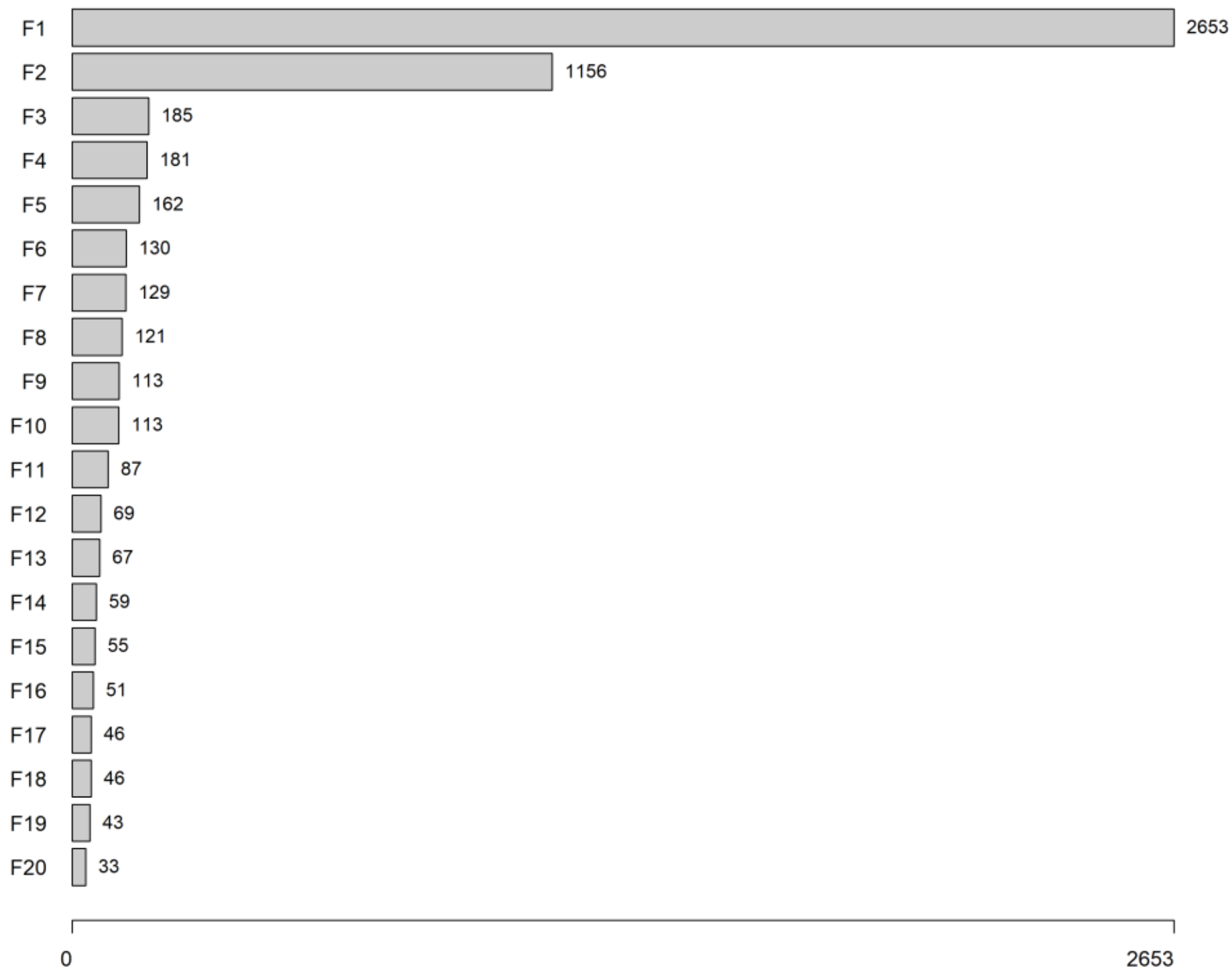

Figure 1. Absolute coefficients of the top 20 features selected by regularized logistic regression from classical radiomics, using inspiratory and expiratory classic radiomic features. Full feature names are listed in Table S2.

Table 1. Table of absolute coefficients from logistic regression based on inspiratory and expiratory features from classic radiomics. Lobar feature suffixes: 1, left upper; 2, left lower; 3, right upper; 4, right middle; 5, right lower; “in” and “ex” indicate inspiration and expiration.

Feature	Name	Coefficient
F1	log-sigma-2-0-mm-3D_gldm_SmallDependenceLowGrayLevelEmphasis_3_in	2653
F2	wavelet-LLL_gldm_SmallDependenceLowGrayLevelEmphasis_2_ex	1156
F3	log-sigma-1-0-mm-3D_glcm_Idmn_4_in	185
F4	log-sigma-3-0-mm-3D_glszm_SmallAreaLowGrayLevelEmphasis_5_ex	181
F5	original_gldm_SmallDependenceLowGrayLevelEmphasis_2_ex	162
F6	log-sigma-2-0-mm-3D_gldm_LowGrayLevelEmphasis_2_ex	130
F7	log-sigma-2-0-mm-3D_glrlm_ShortRunLowGrayLevelEmphasis_2_ex	129
F8	log-sigma-2-0-mm-3D_gldm_LowGrayLevelEmphasis_5_ex	121
F9	wavelet-LHL_glszm_SmallAreaLowGrayLevelEmphasis_5_in	113
F10	log-sigma-2-0-mm-3D_glrlm_LowGrayLevelRunEmphasis_2_ex	113
F11	log-sigma-3-0-mm-3D_gldm_LowGrayLevelEmphasis_5_ex	87
F12	wavelet-LHH_gldm_SmallDependenceLowGrayLevelEmphasis_2_in	69
F13	log-sigma-2-0-mm-3D_glrlm_LowGrayLevelRunEmphasis_5_ex	67
F14	log-sigma-4-0-mm-3D_glcm_Correlation_5_ex	59
F15	wavelet-LLL_glszm_LowGrayLevelZoneEmphasis_2_ex	55
F16	log-sigma-3-0-mm-3D_glrlm_LowGrayLevelRunEmphasis_5_ex	51
F17	log-sigma-5-0-mm-3D_glszm_SmallAreaLowGrayLevelEmphasis_2_ex	46
F18	wavelet-HLH_gldm_SmallDependenceLowGrayLevelEmphasis_1_ex	46
F19	log-sigma-3-0-mm-3D_glrlm_ShortRunLowGrayLevelEmphasis_5_ex	43
F20	wavelet-LHL_glszm_LowGrayLevelZoneEmphasis_5_in	33

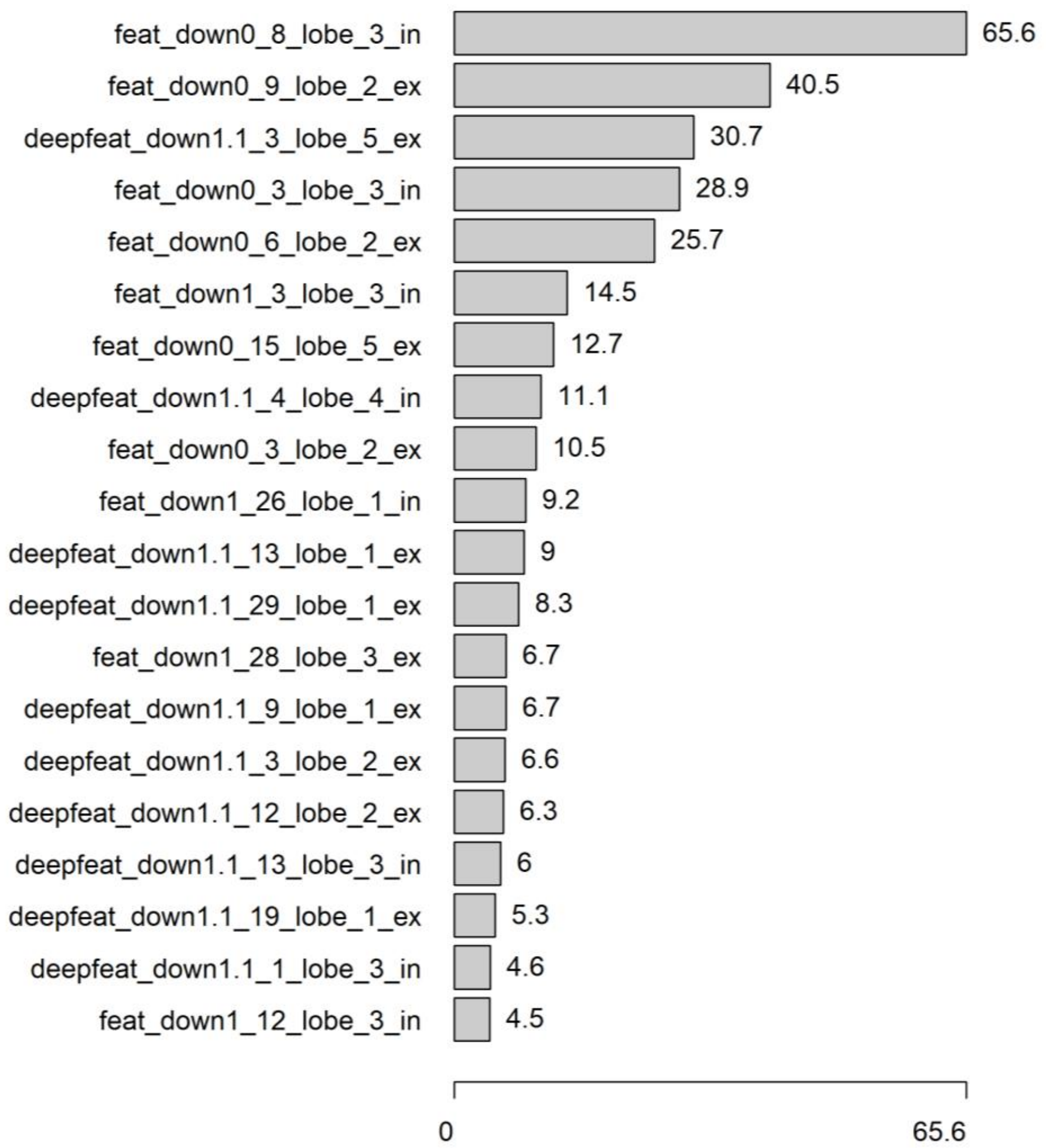

Figure 2. Top 20 features selected by regularized logistic regression using inspiratory and expiratory features from SegResNet. Bars represent the absolute value of the regression coefficients. Lobar feature suffixes: 1, left upper; 2, left lower; 3, right upper; 4, right middle; 5, right lower; “in” and “ex” indicate inspiration and expiration.

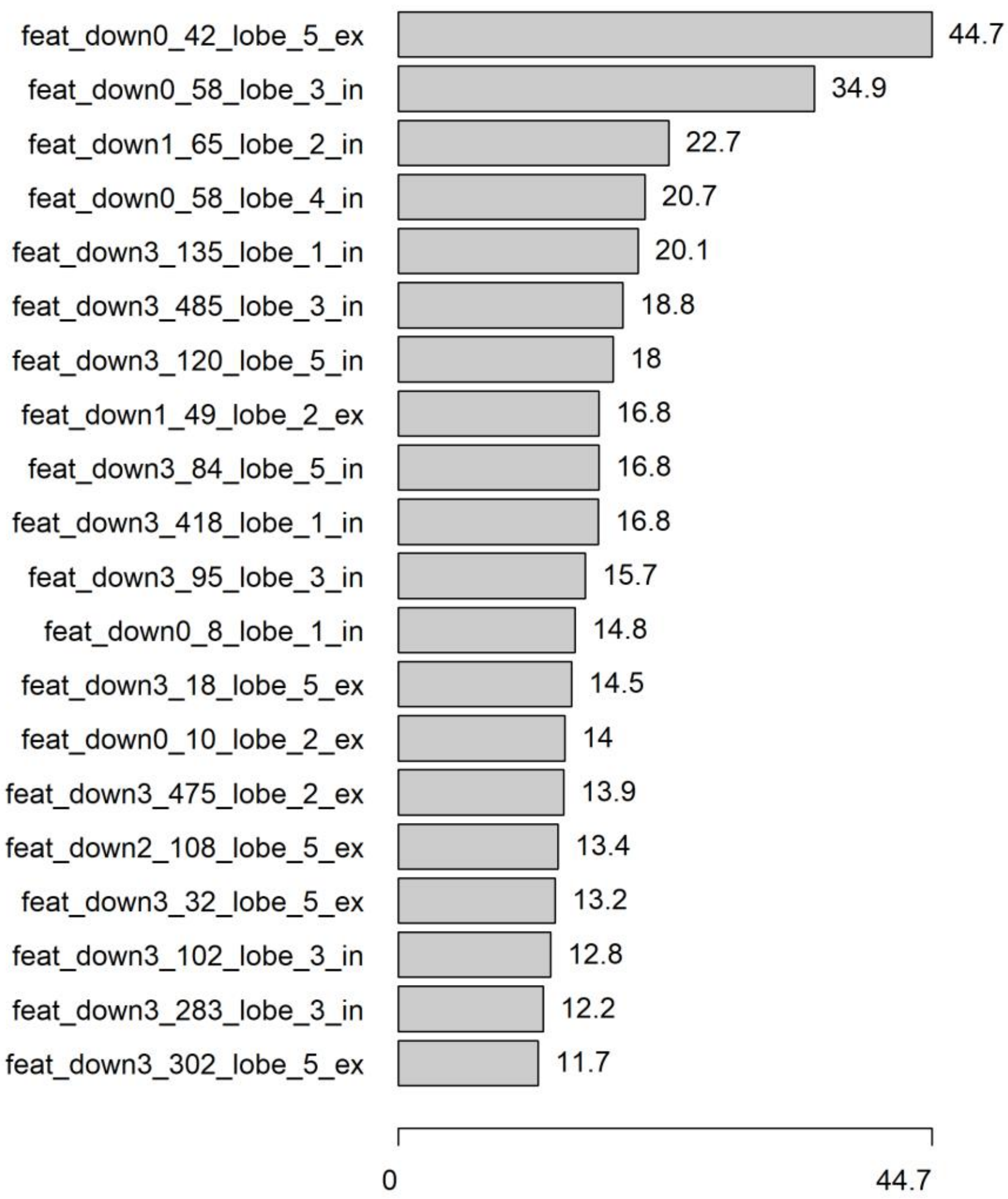

Figure 3. Top 20 features selected by regularized logistic regression using inspiratory and expiratory features from 3D U-Net. Bars represent the absolute value of the regression coefficients. Lobar feature suffixes: 1, left upper; 2, left lower; 3, right upper; 4, right middle; 5, right lower; “in” and “ex” indicate inspiration and expiration.
