# Supplementary material for: Lobar analysis of classical and deep learning radiomic features in chronic airflow limitation
Source: BMC Med Imaging. 2026 Oct 31;26:565. doi: 10.1186/s12880-026-03020-9 (PMC13723113; doi:10.1186/s12880-026-03020-9)
Supplement: Supplementary file 4 [file 12880_2026_3020_MOESM4_ESM.pdf]

Performance of regularized logistic regression models for classification of chronic airflow limitation (CAL): (A) receiver operating characteristic (ROC) curves, (B) precision–recall curves, and (C) calibration curves with Brier scores.

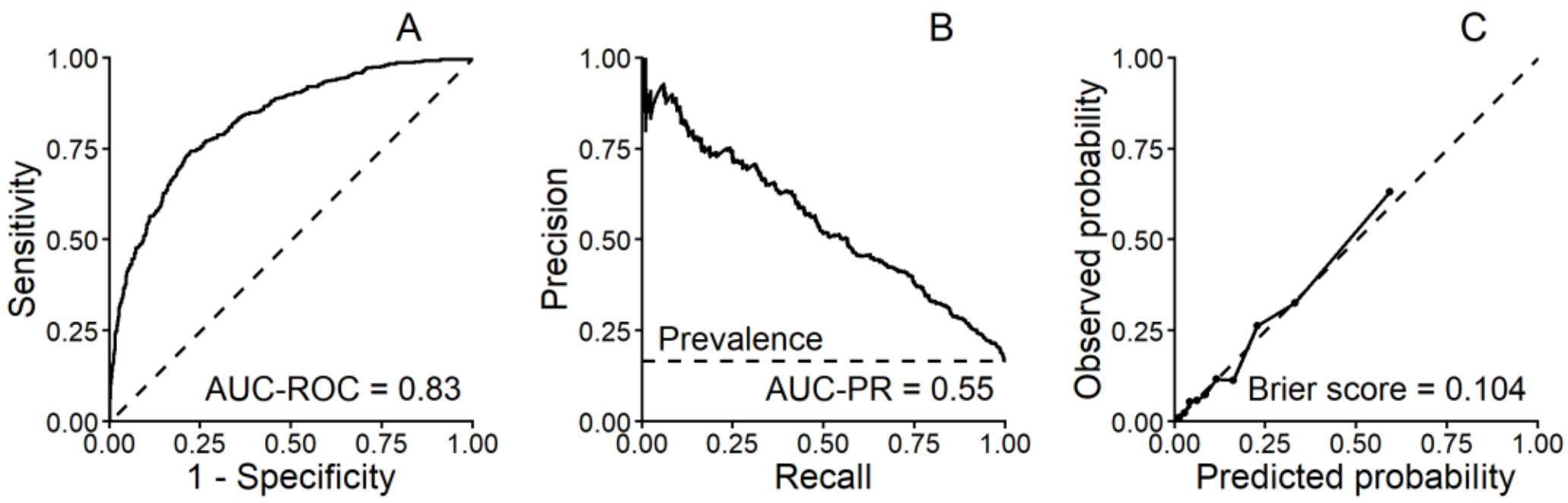

Figure 1. Classical radiomics model, inspiratory features.

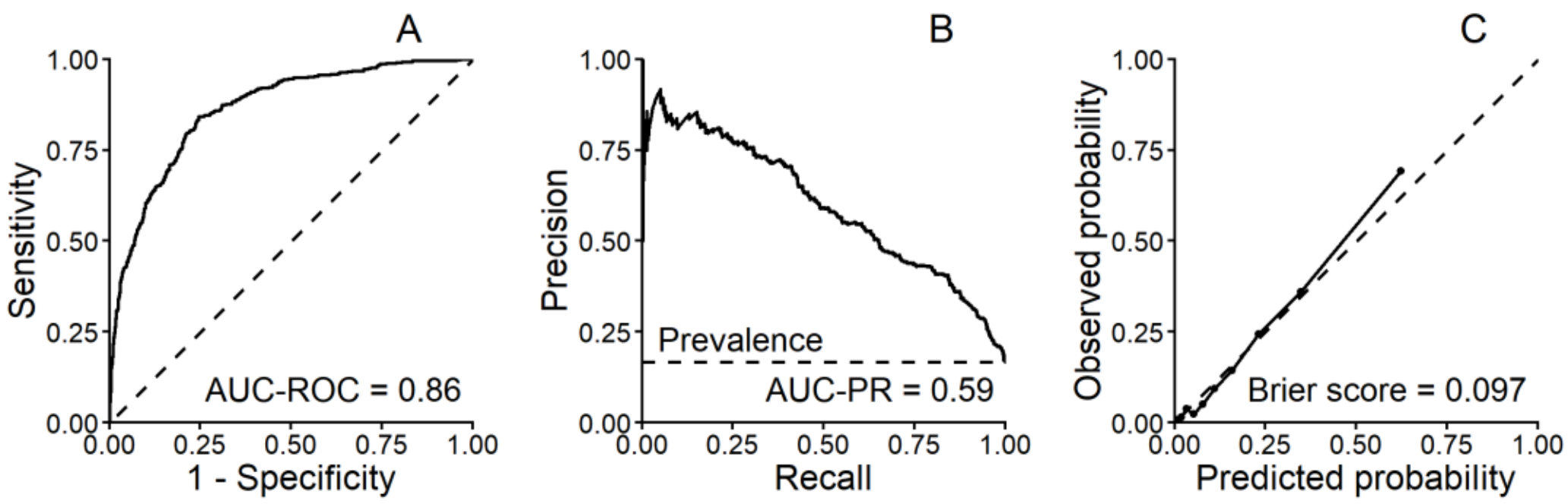

Figure 2. Classical radiomics model, expiratory features.

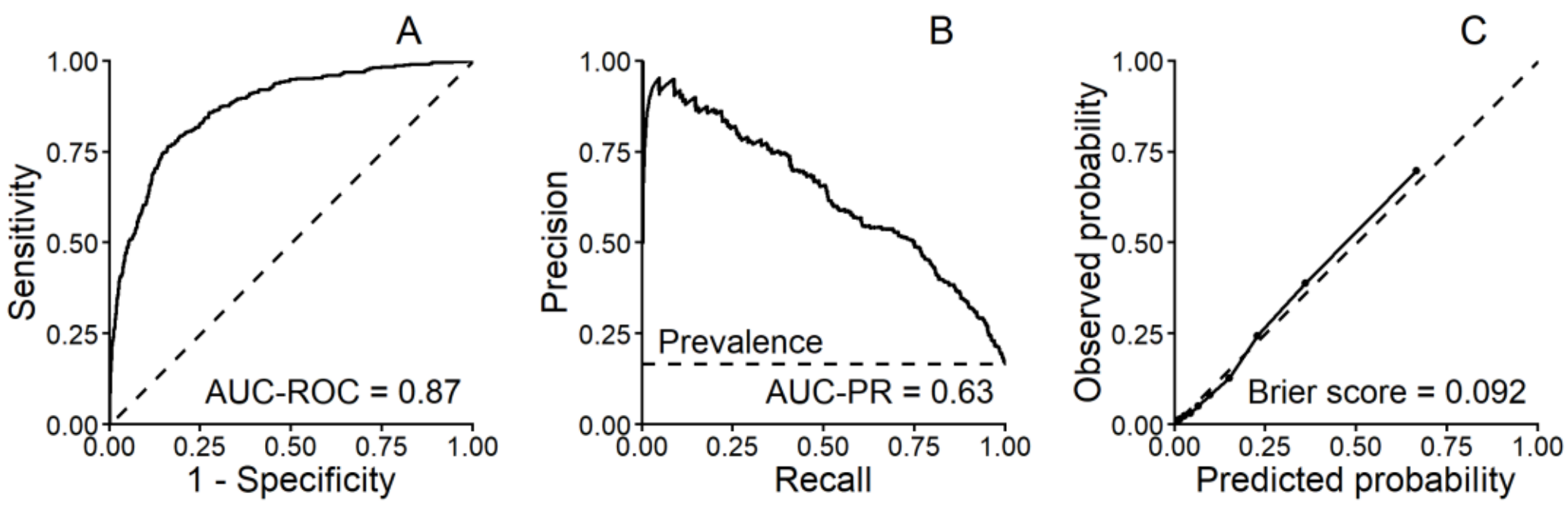

Figure 3. Classical radiomics model, inspiratory and expiratory features.

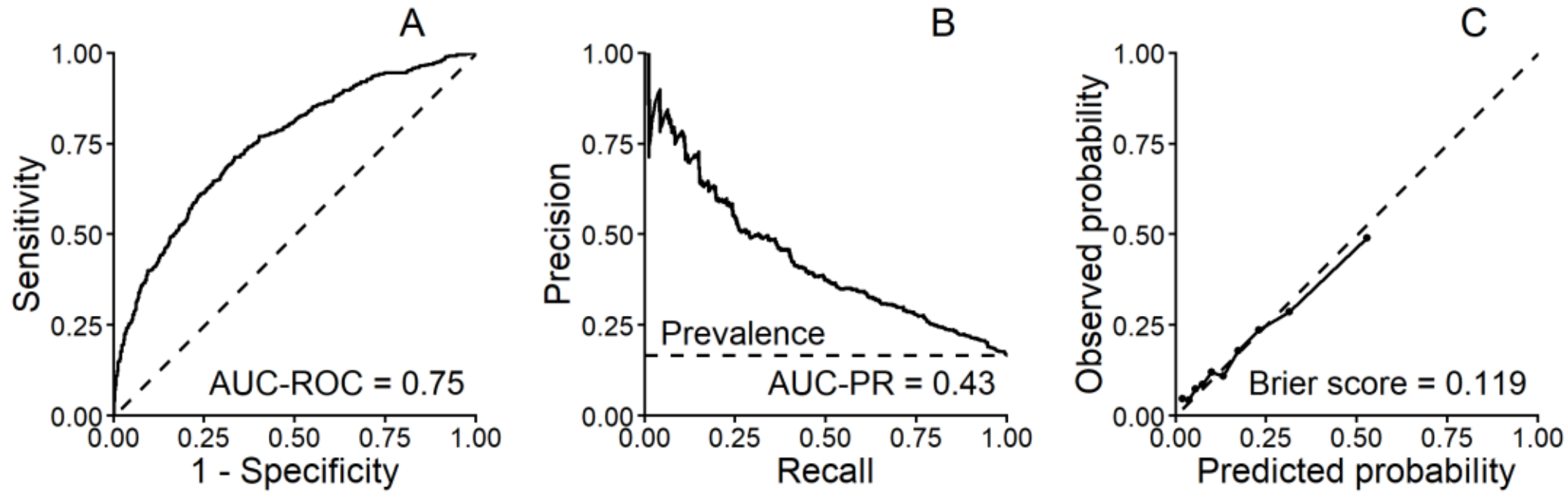

Figure 4. Frozen ResNet transfer-feature model, inspiratory features.

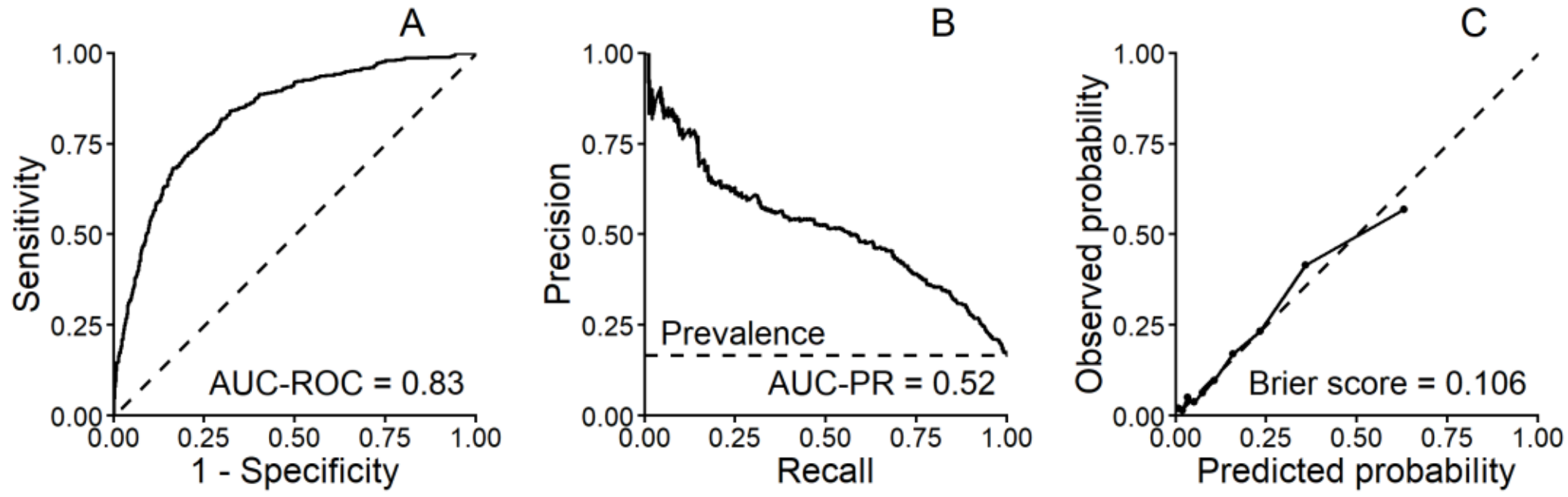

Figure 5. Frozen ResNet transfer-feature model, expiratory features.

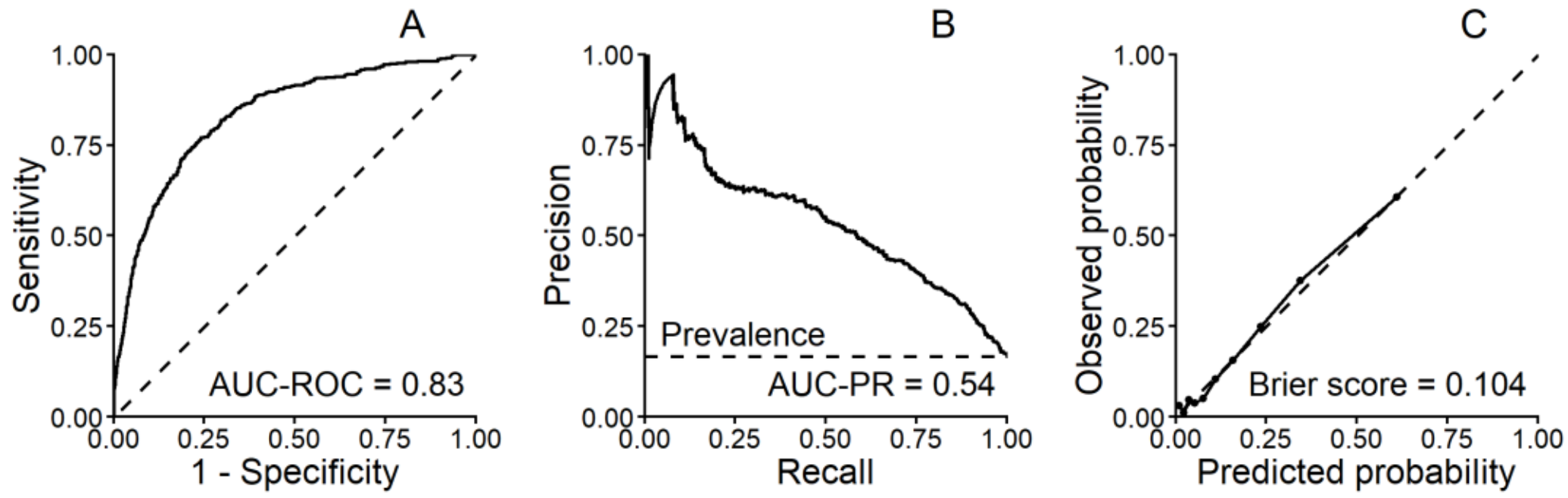

Figure 6. Frozen ResNet transfer-feature model, inspiratory and expiratory features.

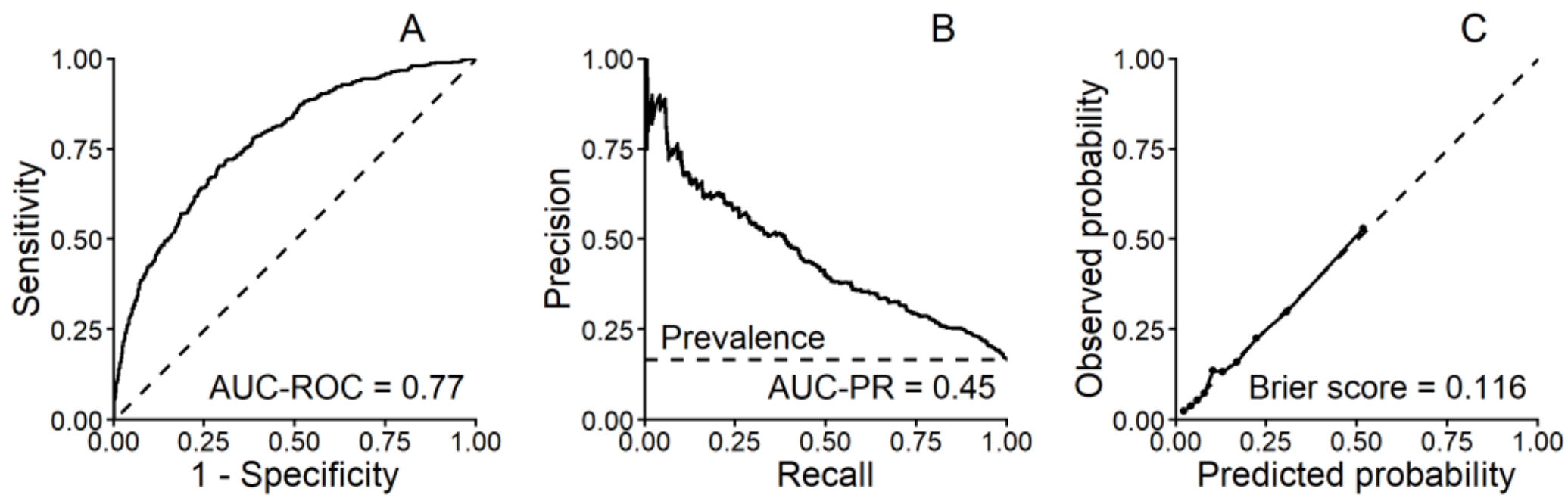

Figure 7. Frozen U-Net transfer-feature model, inspiratory features.

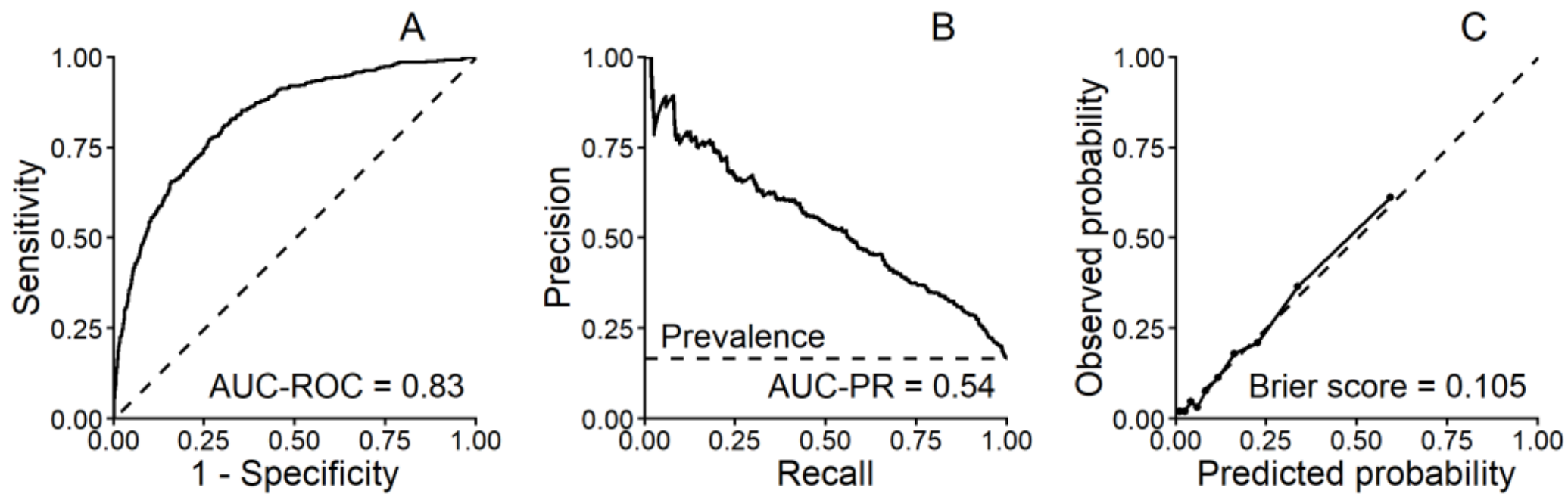

Figure 8. Frozen U-Net transfer-feature model, expiratory features.

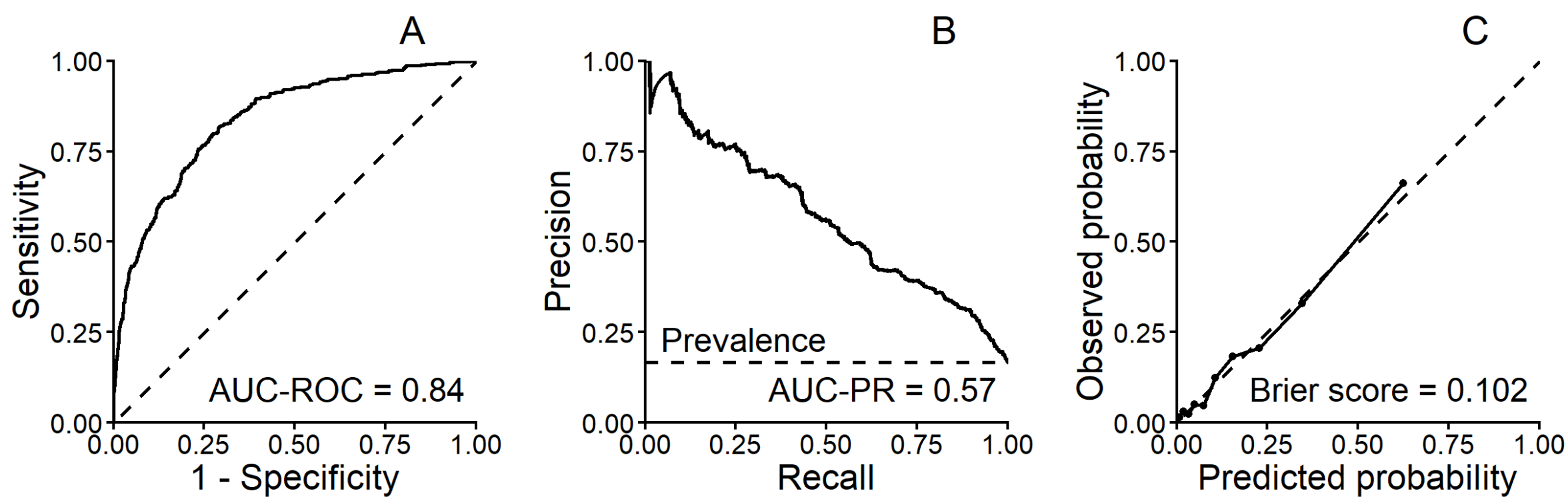

Figure 9. Frozen U-Net transfer-feature model, inspiratory and expiratory features.

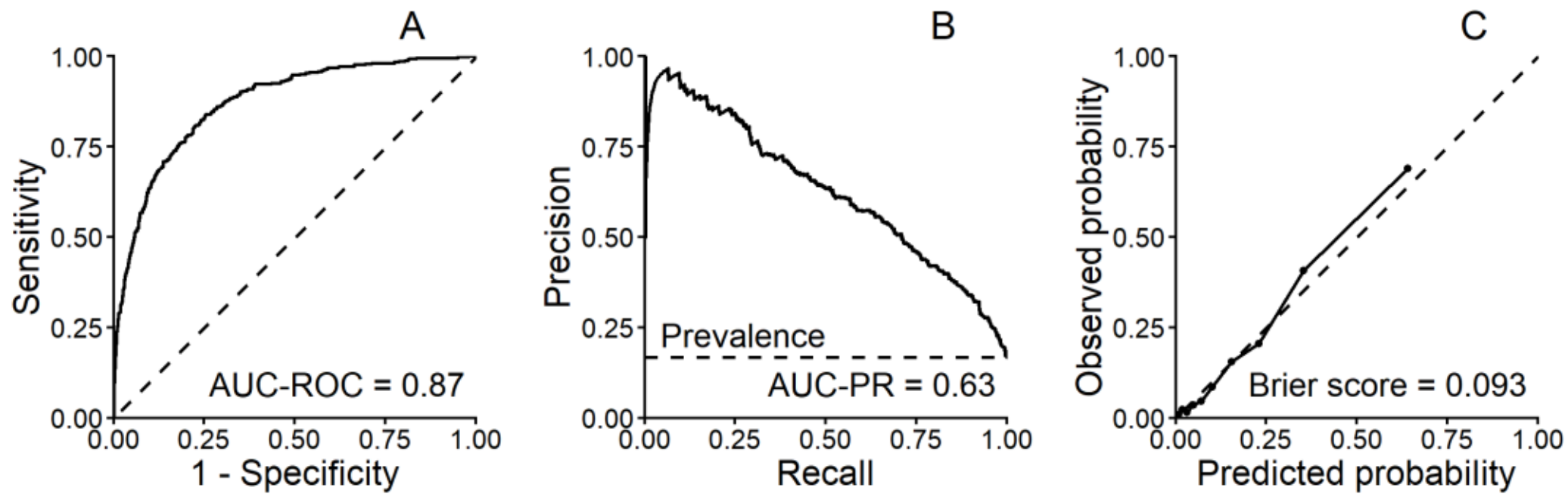

Figure 10. Combined model, classic features and Frozen SegResNet transfer-features, inspiratory and expiratory.

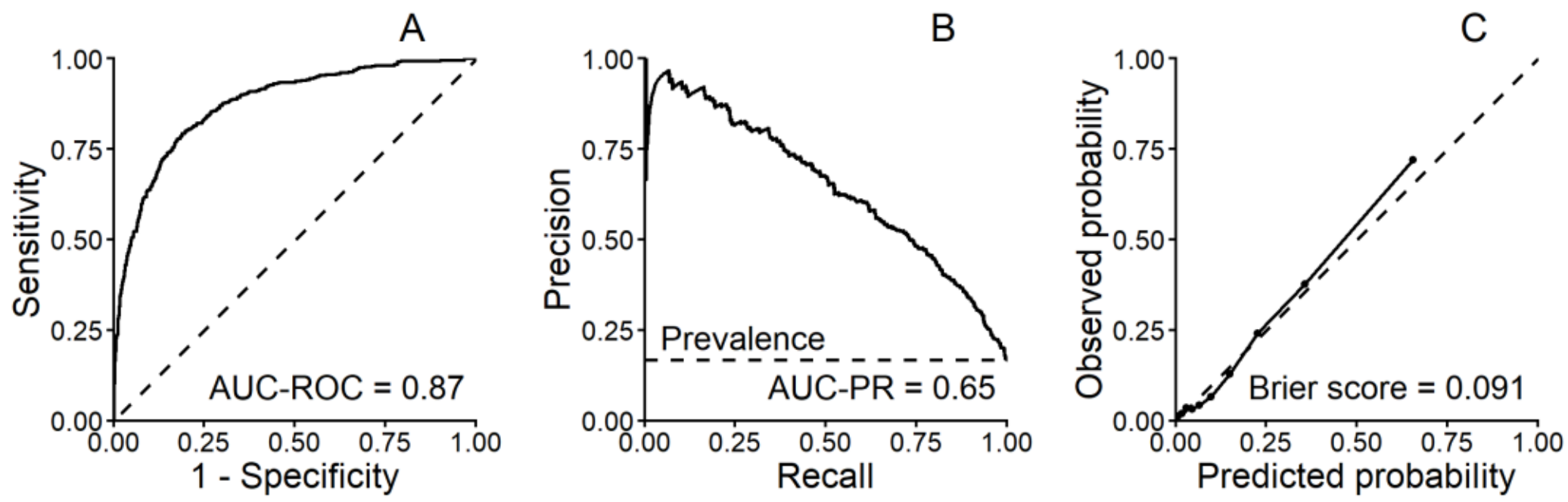

Figure 11. Combined model, classic features and Frozen U-Net transfer-features, inspiratory and expiratory.

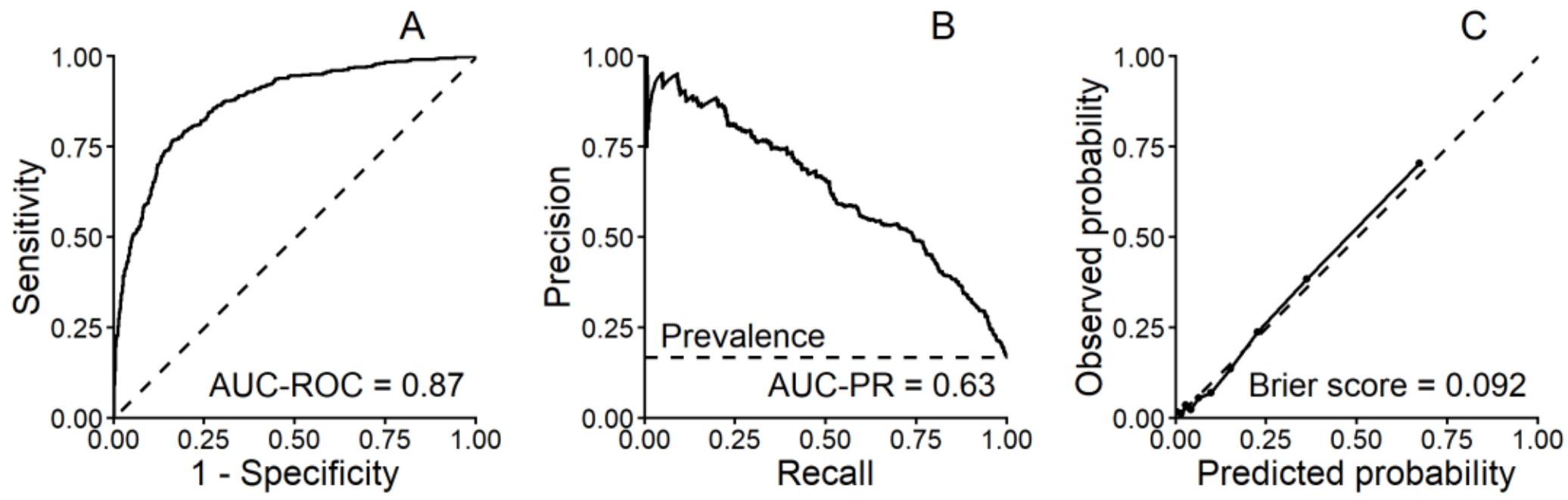

Figure 12. Combined model, classic features and handcrafted features, inspiratory and expiratory.

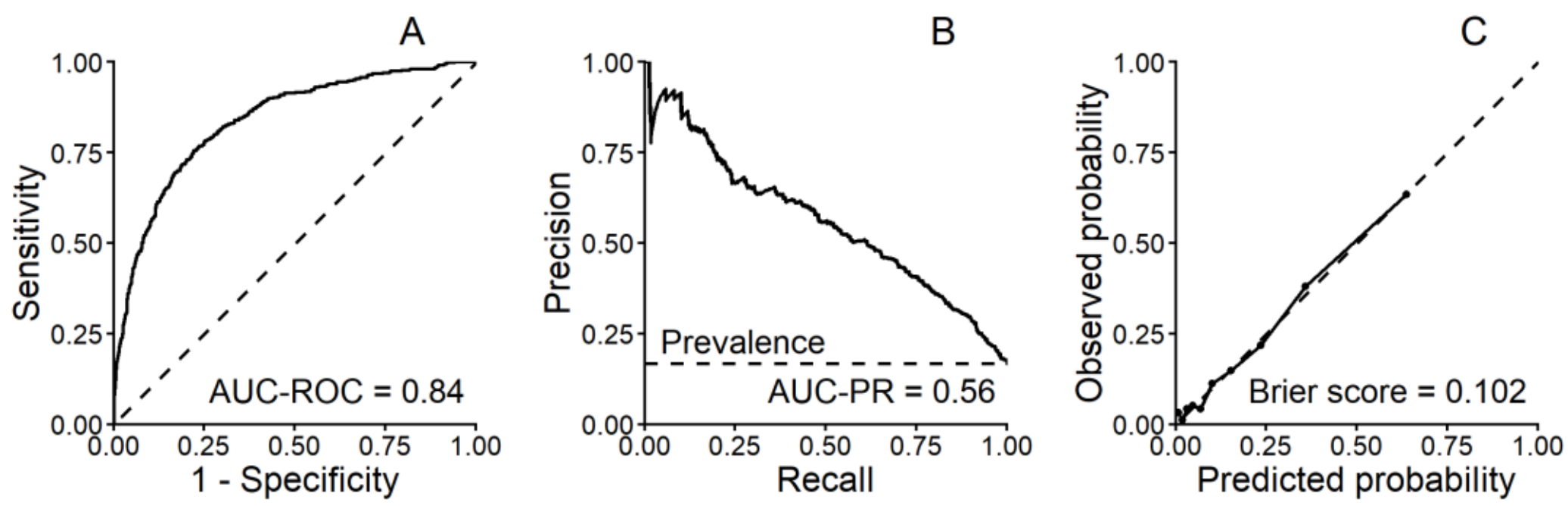

Figure 13. Combined model, frozen SegResNet transfer-features and handcrafted features, inspiratory and expiratory.

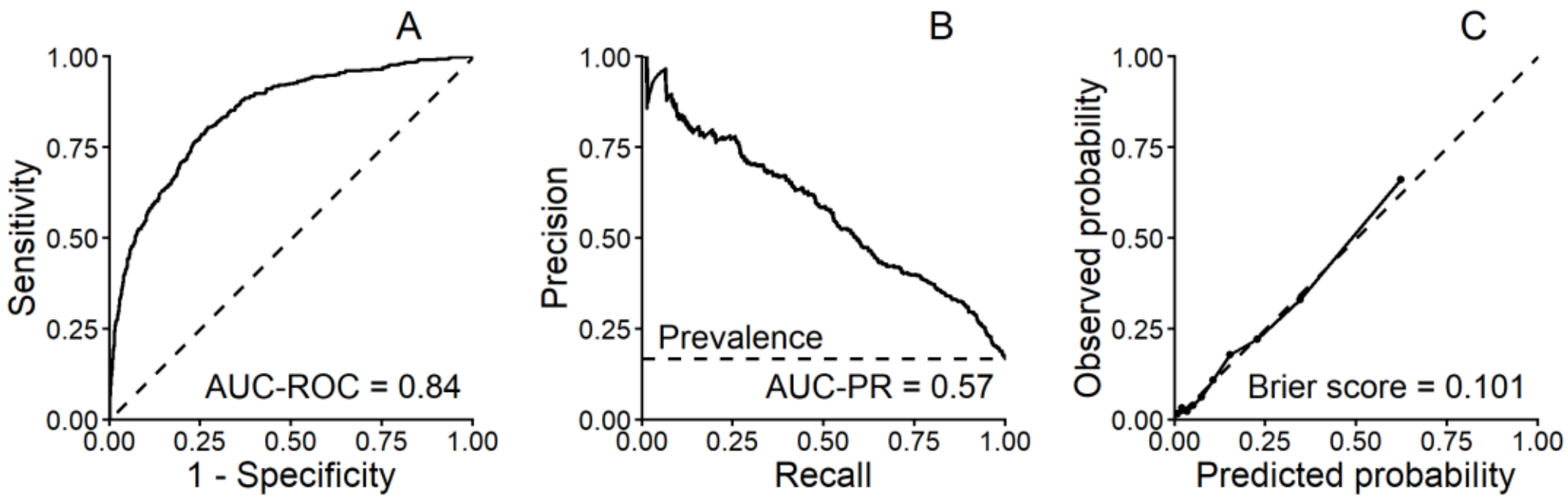

Figure 14. Combined model, frozen U-Net transfer-features and handcrafted features, inspiratory and expiratory.
